# Impact of Cultivar and Grafting on Nutrient and Water Uptake by Sweet Pepper (*Capsicum annuum* L.) Grown Hydroponically Under Mediterranean Climatic Conditions

**DOI:** 10.3389/fpls.2018.01244

**Published:** 2018-08-24

**Authors:** Andreas Ropokis, Georgia Ntatsi, Constantinos Kittas, Nikolaos Katsoulas, Dimitrios Savvas

**Affiliations:** ^1^Laboratory of Vegetable Production, Department of Crop Science, Agricultural University of Athens, Athens, Greece; ^2^Institute of Plant Breeding and Genetic Resources, Hellenic Agricultural Organization – ELGO DEMETER, Thessaloniki, Greece; ^3^Laboratory of Agricultural Constructions and Environmental Control, Department of Agriculture Crop Production and Rural Environment, School of Agricultural Sciences, University of Thessaly, Volos, Greece

**Keywords:** soilless culture, rootstock, calcium, iron, magnesium, bell pepper, scion

## Abstract

In closed-cycle hydroponic systems (CHS), nutrients and water should be delivered to the plants at identical ratios to those they are removed via plant uptake, to avoid their depletion or accumulation in the root zone. For a particular plant species and developmental stage, the nutrient to water uptake ratios, henceforth termed “uptake concentrations” (UC), remain relatively constant over time under similar climatic conditions. Thus, the nutrient to water uptake ratios can be used as nutrient concentrations in the nutrient solution (NS) supplied to CHS to compensate for nutrient and water uptake by plants. In the present study, mean UC of macro- and micronutrients were determined during five developmental stages in different pepper cultivars grown in a closed hydroponic system by measuring the water uptake and the nutrient removal from the recirculating NS. The experiment was conducted in a heated glasshouse located in Athens Mediterranean environment and the tested cultivars were ‘Orangery,’ ‘Bellisa,’ ‘Sondela,’ ‘Sammy,’ self-grafted and ‘Sammy’ grafted onto the commercial rootstock ‘RS10’ (*Capsicum annuum*). ‘Sondela’ exhibited significantly higher NO_3_^-^, Mg^2+^, Ca^2+^ and B UC, while Bellisa exhibited higher K UC in comparison with all other cultivars. The UC of all nutrients were similar in the grafted and the non-grafted ‘Sammy’ plants, which indicates that this *Capsicum annum* rootstock does not modify the uptake of nutrients and water by the scion. The UC of macronutrients estimated in the present study (mmol L^-1^) ranged from 2.4 to 3.7 for Ca, 1.0 to 1.5 for Mg, 6.2 to 9.0 for K, 11.7 to 13.7 for N, and 0.7 to 1.1 for P. The UC of N, K, Ca, and Mg were appreciably higher than the corresponding values found in Dutch tomato glasshouse, while that of P was similar in both locations during the vegetative stage and higher in the present study thereafter. The UC of Fe, Zn and B tended to decrease with time, while that of Mn increased initially and subsequently decreased slightly during the reproductive developmental stage.

## Introduction

In crops grown in closed hydroponic systems (CHS), the net volume of supplied water is essentially equal to that removed via transpiration, if the whole amount of collected drainage solution (DS) is consistently recycled. Furthermore, the input ratio between the mass of a nutrient and the volume of water in a CHS is equal to the concentration of this nutrient in the nutrient solution (NS) supplied to the plants to compensate for nutrient and water uptake by plants. This NS, which is mixed with the DS to be recycled, is commonly termed “nutrient solution for closed systems” (NSCS) (de Kreij et al., 1999). To avoid depletion or accumulation of nutrients in the root zone of a crop grown in a CHS, their concentrations in the NSCS should be equal to the corresponding nutrient to water uptake ratios by the plants, henceforth termed “uptake concentrations” (UC). For a particular plant species and developmental stage, the UC exhibit an appreciable stability over time under similar climatic conditions (Savvas and Lenz, 1995; Sonneveld and Voogt, 2009; Tzerakis et al., 2013). Thus, if the mean UC of all essential nutrients supplied via NS to a particular crop species are known, an appropriate NS composition can be established for the NSCS to be supplied to this crop species (Neocleous and Savvas, 2015).

Standard recommendations for macro- and micro-nutrient levels in NSCS for peppers are mostly based on research carried out in the Netherlands (de Kreij et al., 1999; Sonneveld and Voogt, 2009). However, standard recommendations about the composition of a NSCS for a particular plant species should be based on experimental data originating from similar climatic zones. Indeed, previous research showed that, in crops grown hydroponically under hot and dry climatic conditions such as those prevailing in the Mediterranean basin, the UC may be substantially different than those observed in north-European greenhouses (Neocleous and Savvas, 2015; Savvas et al., 2017). Furthermore, different cultivars, or different rootstocks in the case of grafted plants, may have an impact on nutrient and water uptake, thereby modifying the UC observed in crops of self-rooted plants (Savvas et al., 2010; Rouphael et al., 2016). Indeed, the uptake of nutrients by grafted plants may be influenced not only by the shoot but also by rootstock genotype (Savvas et al., 2017).

To date, in the international scientific literature there is a lack of data about UC arising from experiments with sweet pepper cultivated under hot and dry climatic conditions. Taking this gap of knowledge into consideration, the present study was designed to estimate mean UC for most macro- and micronutrients in a pepper crop grown in a Mediterranean environment, and compare them with similar data arising from north-European climatic conditions. Furthermore, given the high variability of commercial pepper varieties (Tsaballa et al., 2015; Silvar and García-González, 2016), as well as the increasing use of grafted seedlings to establish greenhouse crops of pepper (Penella and Calatayud, 2018), UC were established for four different cultivar types of pepper, one of which was either self-grafted or grafted onto a commercial rootstock. The anticipated data can be used to optimize the composition of NSCS supplied to pepper crops grown in CHS under climatic conditions characterized by mild winters, and dry and hot summers.

## Materials and Methods

### Plant Material and Growth Conditions

The experiment was conducted in a glasshouse at the Agricultural University of Athens (AUA). Four pepper cultivars (*Capsicum annuum* L.) were grown in recirculating NS according to the principles of the Nutrient Film Technique (NFT). Two cultivars of the bell type (Orangery and Sondela) and two of the elongated form (Bellisa and Sammy), one of which (Sammy) was either self-grafted or grafted on a rootstock RS10 (*Capsicum annuum* L.) were used in the experiment. The pepper cultivars chosen for this experiment were the most economically important types of sweet pepper (Bell or elongated form). Grafting was performed only in one sweet pepper cultivar ‘Sammy’ due to the fact that this cultivar was the most commonly grafted cultivar in commercial basis. The rootstock ‘RS10’ was chosen as being the most commonly used in commercial basis and due to the high compatibility with the tested scion. The three non-grafted cultivars and the two versions of ‘Sammy’ i.e., grafted and non-grafted, constituted five experimental treatments. On January 16, 2014, i.e., 2 months after sowing, the seedlings were at the stage of four leaves and had 16 cm mean height, were transferred into 20 closed-loop hydroponic circuits (experimental plots), which were supplied with NS. Each circuit comprised one channel, 3.0 m in length, 0.015 m in width, and 0.03 m in height, which accommodated nine plants. The plant density was 2.5 plants per m^2^. All plants were supplied with a standard NS (de Kreij et al., 1999). Each treatment was replicated four times and thus 20 experimental hydroponic circuits were used. In each experimental unit, each of the 20 experimental units consisted of an individual supply tank, a pump, and irrigation pipes, thereby formed a closed circuit in which a NS was constantly recirculating according to the principles of the NFT. Climatic data during the experiment are given in **Table 1**.

**Table 1:** Monthly averages of temperature (°C) and relative humidity (%) inside the experimental greenhouse.

		January	February	March	April	May	June	July
Temperature (°C)	Average	21.5	22.5	23.8	27.7	29.3	31.3	34.5
	Min	19.0	19.0	19.2	19.3	19.0	19.5	19.5
	Max	23.4	27.0	32.0	34.0	35.5	37.4	39.4
Relative humidity (%)	Average	72.1	71.6	70.6	68.7	62.5	59.1	52.4
	Min	66.3	63.4	58.4	56.8	48.5	44.4	38.8
	Max	74.4	73.6	72.3	70.6	67.8	63.8	56.6


The NS was automatically pumped at a rate of 0.4 m^3^ h^-1^ to each channel while the total volume of the NS that was recirculating in each experimental unit amounted to 3 L per plant. In each unit, the nutrient and water uptake were compensated by automatically supplying a replenishment NS from an individual tank using a floater to maintain a constant NS level in the supply tank. The NS consumed by the plants was counted daily by recording the level difference in the tank filled with replenishment NS. The pH in the recirculating NS was adjusted once per day to 5.6 by adding appropriate amounts of nitric acid to the supply tank based on the actual pH level which was measured using a portable pH-meter. All channels were covered with black–white polyethylene sheets to avoid water evaporation. The total volume of recirculating NS in each experimental unit (including both the solution contained in the supply tank and that flowing in the gullies) amounted to 70 L.

The nutrient concentrations in the NS named as Starter Solution were in all treatments as follows: 6.0 mM K^+^, 6.5 mM Ca^2+^, 2.0 mM Mg^2+^, 0.5 mM NH_4_^+^, 15.6 mM NO_3_^-^, 1.2 mM H_2_PO_4_^-^_,_ 6.5 mM SO_4_^2-^, 15.0 μM Fe, 10.0 μM Mn, 7.0 μM Zn, 0.8 μM Cu, 50.0 μM B, and 0.5 μM Mo. The EC and pH in the above NS were 2.6 dS m^-1^ and 5.6, respectively. After transplanting, the nutrients and water that were absorbed by the plants were replenished daily by supplying replenishment NS with different nutrient concentrations than in the initial NS. The composition of replenishment NS was as follows: Vegetative stage: 5.3 mM K^+^, 3.15 mM Ca^2+^, 1.3 mM Mg^2+^, 1.4 mM NH_4_^+^, 11.6 mM NO_3_^-^, 1.1 mM H_2_PO_4_^-^_,_ 1.2 mM SO_4_^2-^, 15.0 μM Fe, 10.0 μM Mn, 4.0 μM Zn, 0.8 μM Cu, 30.0 μM B and 0.5 μM Mo; Reproductive stage: 6.0 mM K^+^, 2.7 mM Ca^2+^, 1.1 mM Mg^2+^, 0.8 mM NH_4_^+^, 10.6 mM NO_3_^-^, 1.1 mM H_2_PO_4_^-^_,_ 1.1 mM SO_4_^2-^, 15.0 μM Fe, 10.0 μM Mn, 0.7 μM Cu, 5.0 μM Zn, 25.0 μM B, and 0.5 μM Mo. The pH in the recirculating NS was daily adjusted to 5.6–5.7 by adding appropriate amounts of 1 N HNO_3_ stock solution based on the actual pH level which was measured using a portable pH-meter.

### Nutrient Uptake Calculations

Samples of recirculating NS were selected on a 4-weekly basis from all experimental units to determine the actual Ca, Mg, K, P, B, NO_3_, Fe, Mn, and Zn concentrations. The concentrations of Ca, Mg, Fe, Mn, and Zn in both the NSs and the aqueous extracts of plant tissues were measured using an atomic absorption spectrophotometer (Perkin Elmer 1100A, Perkin Elmer, Waltham, MA, United States) while K was determined by flame photometry (Sherwood Model 410, Cambridge, United Kingdom). The NO_3_^-^, P and B concentrations in NS samples were measured by UV/VIS spectroscopy at 540, 880, and 420 nm, respectively.

Nutrient to water uptake ratios (uptake concentrations) for K, Mg, Ca, and Fe were estimated based on the removal of nutrients from the NS. In particular, the mean uptake concentration of the *x* micronutrient (*C_xu_* in μmol L^-1^, where *x* = Ca, Mg, K, P, B, NO_3_, Fe, Mn, and Zn) was determined for four time intervals using the following mass balance equation:

$$Cxu=\frac{Vr\left(Cxbi-Cxei\right)+VuiCxa}{Vui}$$

where *V_r_* (L) denotes the total volume of the recirculating NS in each experimental unit (70 L in the present study), *V_ui_* (L) denotes the total volume of NS that was taken up by the plants in each experimental unit during the *i* time interval (*i* = 1…5), *C_xbi_* and *C_xei_* (μmol L^-1^) denote the concentrations of the *x* nutrient in the recirculating NS on the first and the last day of the *i* time interval (*i* = 1…5), and *C_xa_* (μmol L^-1^) denotes the concentration of the *x* nutrient in the replenishment NS used in each treatment.

### Statistical Analysis

All data were statistically analyzed by applying ANOVA using the PlotIT3.2^®^ software package, version 3.2 for Windows (Scientific Programming Enterprises, Haslett, MI, United States). Data are presented in graphs as means ± SE.

## Results

**FIGURE 1:**
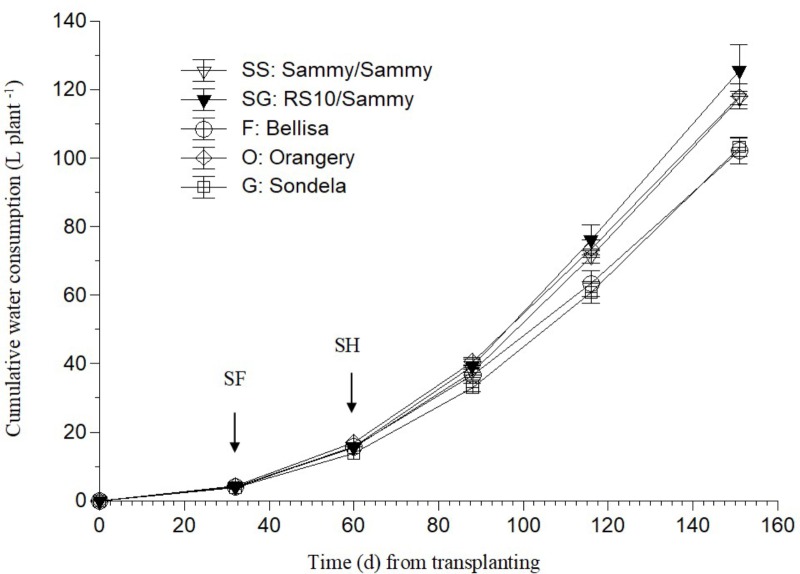
Evolution of the cumulative water consumption (CWC) in a pepper crop grown in a closed hydroponic system as influenced by four different pepper cultivars (‘Orangery,’ ‘Bellisa,’ ‘Sondela,’ ‘Sammy’) and grafting (‘Sammy’ self-grafted or grafted onto the commercial rootstock ‘RS10’). Vertical bars indicate ± standard errors of means. SF, start of flowering; SH, start of harvesting.

As shown in **Figure 1**, ‘Bellisa’ and ‘Sondela’ consumed about 15% less water than ‘Orangery’ and ‘Sammy’ self-grafted, and about 20% less than Sammy grafted onto the commercial rootstock RS10. These differences reflected commensurate differences in the leaf area and the vegetative plant biomass (data not shown). However, as shown in **Table 2**, the differences in water consumption and vegetative plant biomass had no significant impact on the total fruit production, although ‘Orangery’ rendered a slightly higher yield than the other cultivars. In contrast to the total fruit yield, the number of fruit per plant and the mean fruit weight were strongly influenced by the genotype. In particular, ‘Orangery’ and ‘Sondela’ produced much less fruit than ‘Sammy’ but the weight of fruit from the latter cultivar was much smaller. As a result, the total yield was similar in all cultivars without any significant differences between them. On the other hand, hetero-grafting of ‘Sammy’ onto the commercial rootstock RS10 had no impact on the total fruit yield, the number of fruit per plant or the mean fruit weight. Due to the similar yield performance and the significantly lower cumulative water consumption in comparison to ‘Sammy’ and ‘Belisa,’ ‘Sondela’ exhibited a significantly higher water use efficiency (WUE) than these two cultivars (**Table 2**). However, the WUE of ‘Orangery’ was as high as that of ‘Sondela.’ It is worth to mention that grafting onto the commercial *Capsicum annuum* rootstock ‘RS10’ did not improve the WUE of ‘Sammy.’

**Table 2:** Influence of four cultivated varieties.

Grafting combination (rootstock/scion)	WUE (g L^-1^)	TFW/plant (kg)	TFN/plant (No)	MFW/plant (g)
‘Sammy’/‘Sammy’	28.3 b	3.36	46.00 a	72.94 b
‘RS10’/‘Sammy’	24.9 c	3.14	48.25 a	65.16 b
‘Bellisa’	29.0 b	3.05	38.75 b	78.74 b
‘Orangery’	33.1 a	3.97	26.25 cd	151.13 a
‘Sondela’	33.3 a	3.44	23.33 d	147.31 a
*Statistical significance*		
Grafting combination	^∗^	ns	^∗∗^	^∗∗∗^


*‘Orangery’ (O), ‘Bellisa’ (F), ‘Sondela’ (G), and ‘Sammy’ self-grafted (SS) or ‘Sammy’ grafted onto a commercial rootstock ‘RS10’ (SG), grown in closed hydroponic systems, on total fruit weigh (TFW) per plant, total fruit number (TFN) per plant, and average marketable fruit weigh (MFW) per plant. ns, ^∗^, ^∗∗^, and ^∗∗∗^ not significant or significant at *p* ≤ 0.05, 0.01, and 0.001, respectively. Mean (*n* = 3) followed by different letters within the same column indicate significant differences for each factor according to the Duncan’s multiple range test.*

**FIGURE 2:**
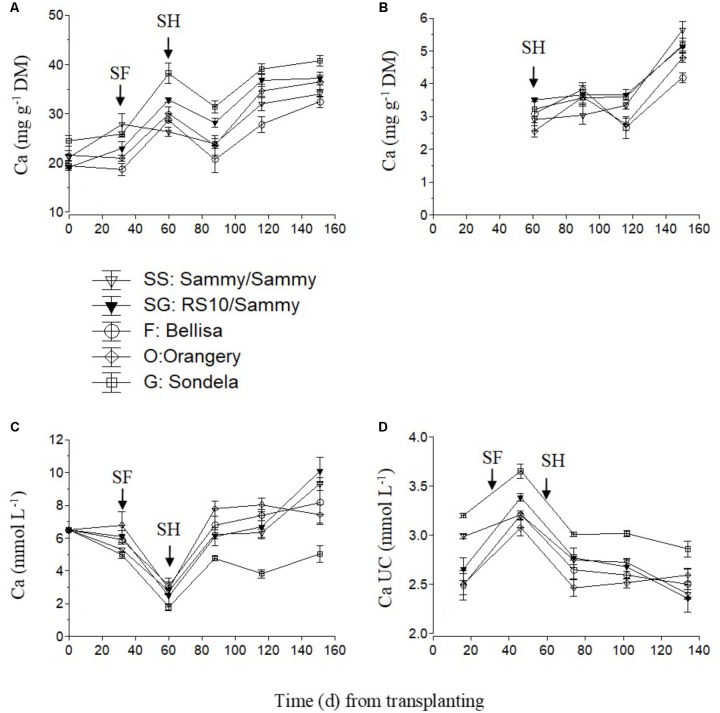
Impact of different pepper cultivars (‘Orangery,’ ‘Bellisa,’ ‘Sondela,’ ‘Sammy’) and grafting (‘Sammy’ self-grafted or grafted onto the commercial rootstock ‘RS10’) on: (i) concentrations of Ca in the dry mass (DM) of young leaves **(A)** and fruit **(B)**, (ii) concentrations of Ca in the recirculating nutrient solution **(C)**, and (iii) apparent Ca uptake concentrations (UC), i.e., mmol of Ca uptake per L of water uptake **(D)**. Vertical bars indicate ± standard errors of means. SF, start of flowering; SH, start of harvesting.

The Ca concentration in the recirculating NS was appreciably reduced in the time between the start of anthesis and the start of harvesting (**Figure 2C**), because the uptake of Ca per L of water was maximized (**Figure 2D**), irrespective of the cultivar. The increased Ca uptake during this developmental stage resulted in higher leaf Ca concentrations at commencement of harvesting in comparison with the vegetative growth stage (**Figure 2A**). After commencement of harvesting, the Ca UC tended to decrease gradually (**Figure 2D**), and this resulted in increased Ca levels in the recirculating NS, although the leaf Ca concentrations did not decrease. The fruit Ca concentrations increased during the late cultivation period, regardless of the root and shoot genotype (**Figure 2B**).

**FIGURE 3:**
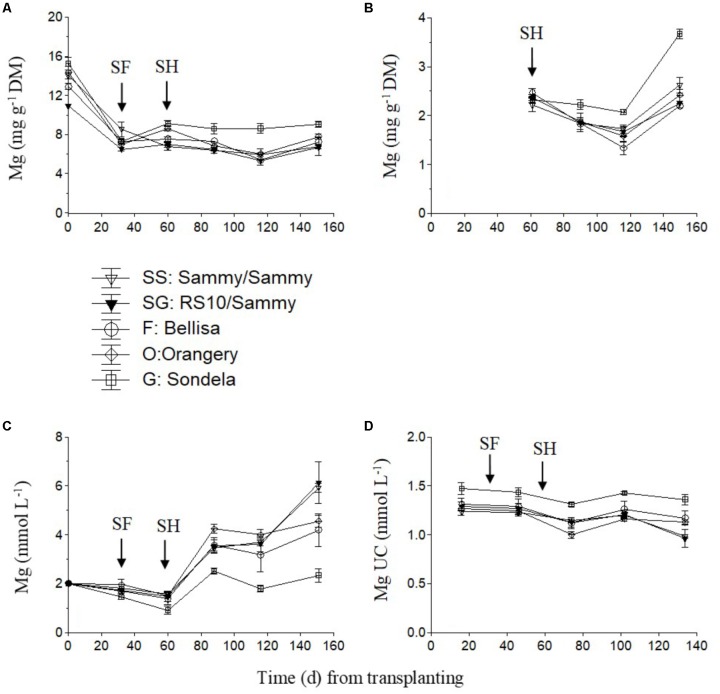
Impact of different pepper cultivars (‘Orangery,’ ‘Bellisa,’ ‘Sondela,’ ‘Sammy’) and grafting (‘Sammy’ self-grafted or grafted onto the commercial rootstock ‘RS10’) on: (i) concentrations of Mg in the dry mass (DM) of young leaves **(A)** and fruit **(B)**, (ii) concentrations of Mg in the recirculating nutrient solution **(C)**, and (iii) apparent Mg uptake concentrations (UC), i.e., mmol of Mg uptake per L of water uptake **(D)**. Vertical bars indicate ± standard errors of means. SF, start of flowering; SH, start of harvesting.

The Mg concentration in the recirculating NS tended to decrease slightly up to the start of harvesting but thereafter it showed an increasing tendency which was very weak in ‘Sondela’ and stronger in ‘Sammy,’ regardless of the root genotype (**Figure 3C**). On the other hand, the highest leaf Mg concentrations (**Figure 3A**) and Mg UC (**Figure 3D**) were measured in ‘Sondela’ throughout the cropping period. The leaf Mg concentrations were significantly higher at the fully vegetative stage (immediately after planting) than at later developmental stages, regardless of the root and shoot genotype. After commencement of flowering, both the leaf Mg concentrations and the Mg UC (**Figure 3D**) remained more or less constant during the whole cropping period. The fruit Mg concentrations tended to decrease up to day 120 from treatment initiation but this tendency was reversed at the late growing period. ‘Sondela’ exhibited consistently higher fruit Mg concentrations after commencement of harvesting in comparison with the other cultivars, while grafting had no impact on the fruit Mg level (**Figure 3B**).

**FIGURE 4:**
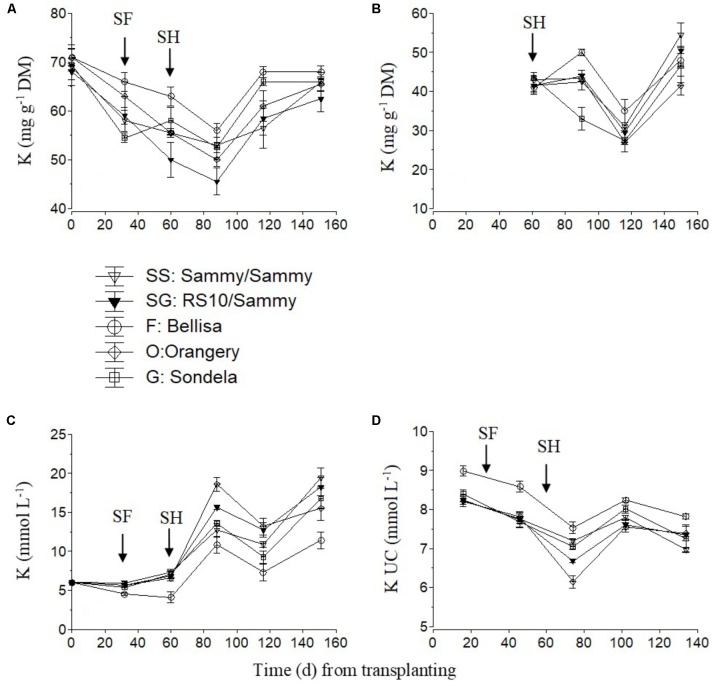
Impact of different pepper cultivars (‘Orangery,’ ‘Bellisa,’ ‘Sondela,’ ‘Sammy’) and grafting (‘Sammy’ self-grafted or grafted onto the commercial rootstock ‘RS10’) on: (i) concentrations of K in the dry mass (DM) of young leaves **(A)** and fruit **(B)**, (ii) concentrations of K in the recirculating nutrient solution **(C)**, and (iii) apparent K uptake concentrations (UC), i.e., mmol of K uptake per L of water uptake **(D)**. Vertical bars indicate ± standard errors of means. SF, start of flowering; SH, start of harvesting.

The K concentration in the recirculating NS tended to increase slightly after the start of flowering and strongly after commencement of harvesting and up to 85 days after planting (**Figure 4C**). This increase in the recirculating NS was combined with a decrease in the leaf K concentration (**Figure 4A**) and the K UC (**Figure 4D**). Nevertheless, thereafter, both the leaf K concentration and the K UC increased again to similar levels with those measured in the interval between commencement of flowering and harvesting. Bellisa exhibited consistently higher UC than the other cultivars throughout the cropping period and this resulted in lower K concentrations in the recirculating NS and higher leaf K concentrations after the start of flowering. In contrast, ‘Orangery’ exhibited lower K UC after the start of harvesting but the differences were not always significant. The fruit K concentrations tended to increase slightly after commencement of harvesting and up to 85 days after treatment initiation, with the exception of ‘Sondela’ and ‘Bellisa’ which exhibited lower and higher fruit K concentrations, respectively, than the other cultivars, although this was not consistent during the whole cropping period (**Figure 4B**).

**FIGURE 5:**
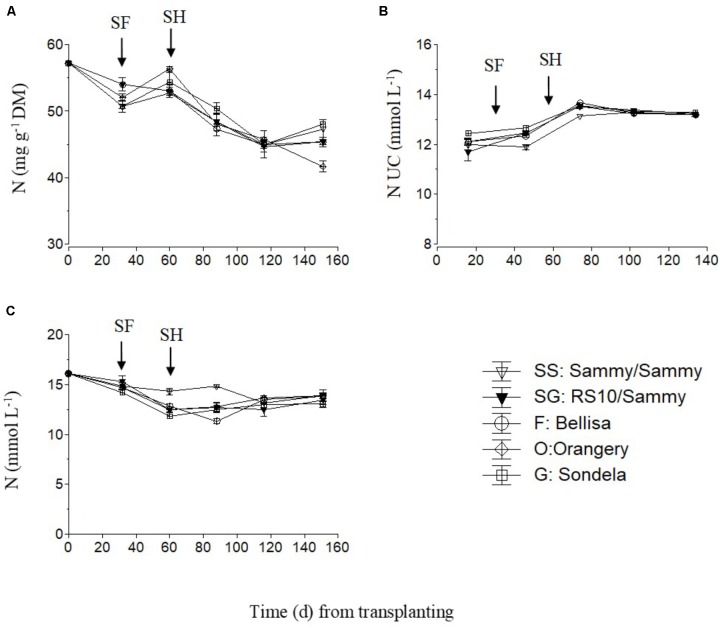
Impact of different pepper cultivars (‘Orangery,’ ‘Bellisa,’ ‘Sondela,’ ‘Sammy’) and grafting (‘Sammy’ self-grafted or grafted onto the commercial rootstock ‘RS10’) on: (i) concentrations of total-N in the dry mass (DM) of young leaves **(A)**, (ii) apparent total-N uptake concentrations (UC), i.e., mmol of total-N uptake per L of water uptake **(B)**, and (iii) concentrations of total-N in the recirculating nutrient solution **(C)**. Vertical bars indicate ± standard errors of means. SF, start of flowering; SH, start of harvesting.

The total nitrogen (NO_3_-N and NH_4_-N) concentrations in the recirculating NS solution (**Figure 5C**) and the leaves of pepper (**Figure 5A**) showed a slight decreasing tendency during the cropping period, while the total-N UC followed an increasing course up to the commencement of harvesting and shortly thereafter (**Figure 5B**). However, the total-N UC remained constant during the harvesting period. The self-grafted ‘Sammy’ plants tended to take up less N per L of water after the start of flowering and at the very early stage of harvesting but thereafter the N UC were not influenced by the pepper cultivar. The fruit N concentrations were not measured due to a technical failure which resulted in impairment of the samples.

**FIGURE 6:**
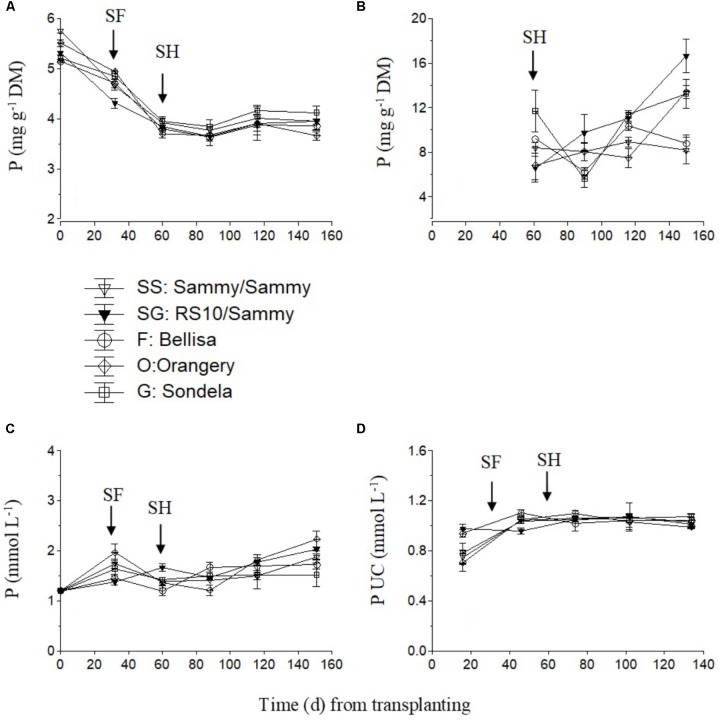
Impact of different pepper cultivars (‘Orangery,’ ‘Bellisa,’ ‘Sondela,’ ‘Sammy’) and grafting (‘Sammy’ self-grafted or grafted onto the commercial rootstock ‘RS10’) on: (i) concentrations of P in the dry mass (DM) of young leaves **(A)** and fruit **(B)**, (ii) concentrations of P in the recirculating nutrient solution **(C)**, and (iii) apparent P uptake concentrations (UC), i.e., mmol of P uptake per L of water uptake **(D)**. Vertical bars indicate ± standard errors of means. SF, start of flowering; SH, start of harvesting.

The P concentration in the recirculating NS showed an increasing tendency in the interval from planting up to the start of flowering in self-grafted ‘Sammy,’ ‘Orangery,’ and ‘Sondela’ (**Figure 6C**) which was accompanied by a commensurate decrease in the P UC at the same time interval (**Figure 6D**). In contrast, the P UC and the P levels in the recirculating NS of ‘Sammy’ grafted onto RS10 and ‘Belisa’ did not change by the time of flowering commencement in comparison to the initial vegetative growth stage. After the start of flowering, both the P concentration in the recirculating NS and the P UC remained more or less constant up to crop termination. Nevertheless, the leaf P concentration showed a consistent tendency to decrease after planting in all cultivars, which lasted up to the start of flowering (**Figure 6A**). The fruit P concentrations did not differ significantly between the different experimental treatments while it exhibited no consistent tendency during the cropping period (**Figure 6B**).

**FIGURE 7:**
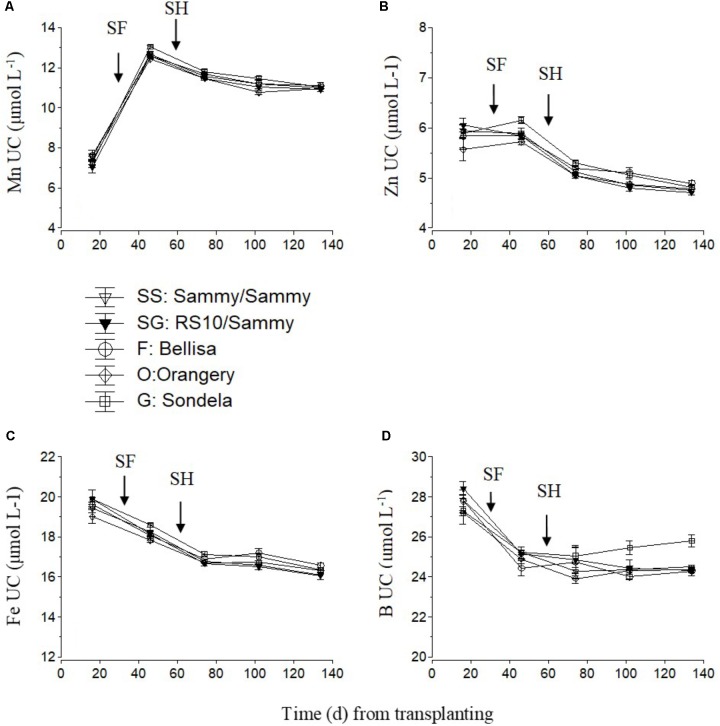
Estimated apparent uptake concentration of micro-nutrient (UC), (i.e., mmol of micro-nutrient uptake per L of water uptake), as influenced by four cultivated varieties. i.e., ‘Orangery’ (O), ‘Bellisa’ (F), ‘Sondela’ (G), and ‘Sammy’ self-grafted (SS) or ‘Sammy’ grafted onto a commercial rootstock ‘RS10’ (SG). UC was calculated on the basis of **(A)** manganese (Mn), **(B)** zinc (Zn), **(C)** iron (Fe), and **(D)** boron **(B)** and water removal from the recirculating nutrient solution. Vertical bars indicate ± standard errors of means. SF, start of flowering; SH, start of harvesting.

For micronutrients, only the UC are shown (**Figure 7**). The UC of Mn and Zn were highest during the vegetative growth stage and decreased slightly but constantly after commencement of harvesting. No consistent differences in UC between the different cultivars were found, although ‘Orangery’ showed a lower UC than the other cultivars during the vegetative stage (**Figure 7A**) and ‘Sondela’ showed a higher UC than the other cultivars (**Figure 7B**) in the interval between the start of flowering and the start of harvesting. The Fe UC also decreased constantly during the cropping period from 19.5 to 16 μmol L^-1^ on average, without any significant differences between the tested cultivars (**Figure 7C**). The B UC decreased from 27.6 μmol L^-1^ on average during the vegetative growth stage to about 25 μmol L^-1^ after the start of harvesting and remained roughly constant to these levels during the reproductive stage (**Figure 7D**). So significant differences between treatments could be found during the vegetative stage of growth, while during the late reproductive stage (after the first 3 months from planting) the B UC of ‘Sondela’ was significantly higher than those of the other tested cultivars. Grafting of ‘Sammy’ onto the rootstock RS10 had no significant impact on the UC of micronutrients.

## Discussion

The present study showed that the developmental stage of the pepper plants has a strong impact on the nutrient to water uptake ratios (UC). For most nutrients, i.e., K, Ca, Mg, and all micronutrients studied, the UC tended to decrease with time and only the UC of total-N and P increased slightly after the initial vegetative stage. By definition, the UC (nutrient to water uptake ratios) depend on two variables, i.e., the rates of nutrient uptake and the rates of water uptake, which are independent to each other, since the plant metabolism is physiologically not related to transpiration (Taiz and Zeiger, 2002). Thus, any difference in the UC at different plant developmental stages or between different cultivars may be due to commensurate differences either in the nutrient uptake rates, or in the water uptake rates, or in both. As the plants are growing up, the leaf area increases and this results in increased rates of whole plant transpiration (Taiz and Zeiger, 2002). On the other hand, an increased leaf area results in increased rates of whole plant photosynthesis (Koyama and Kikuzawa, 2009), thereby raising the nutrient demand and concomitantly the nutrient uptake rates. As a result, although the nutrient uptake and the water uptake are independent processes, the UC do not change dramatically during the cropping period, as has been shown in several studies (Savvas and Lenz, 1995; Sonneveld and Voogt, 2009). However, morphological changes at different plant developmental stages may differently affect the nutrient uptake rates than the water uptake rates. Thus, at the early plant developmental stages, all or most leaves are young, while at later developmental stages, when the plants enter the reproductive phase, a considerable part of the foliage consist of older leaves (Paltridge and Denholm, 1974). The rates of transpiration are much less affected by the age of the leaves than the rates of net photosynthesis. Consequently, as the age of the plants increases, the water uptake rates increase more than the nutrient uptake rates and thus the UC decrease, although in absolute terms the nutrient demand increases. The tendency of the UC for most nutrients (except of N and P) to decrease with time in the present study seems to result mainly from a stronger increase in water uptake rates with time than in the nutrient uptake rates.

The exceptions formed by the UC of N and P, which increased slightly as the plants entered the reproductive phase, may indicate a stronger increase in the N and P demand during the reproductive stage than the increase in the whole plant transpiration. An increase in the P demand by pepper as the plants pass from the vegetative to the reproductive stage can be ascribed to the appreciably higher P concentrations in the fruit than in the leaves of pepper. Indeed, as reported by Silber et al. (2005), the P concentrations in the leaves of pepper ranged from 2.1 to 3.7 mg g^-1^, depending on irrigation frequency and P application rate, while the corresponding values in the fruit ranged from 6.2 to 7.5 mg g^-1^. Similar differences in the P concentration between leaves and fruit were found also in the present study (**Figure 6**). However, the nitrogen concentrations tend to be higher in the leaf than in the fruit (e.g., 48.6 vs. 37.7 mg g^-1^, respectively, as reported by Bar-Tal et al., 2001). Hence, the slight increase in the N UC as the plants entered the reproductive stage in the present study cannot be ascribed to increased fruit N concentrations compared to those in leaves. An alternative explanation is a possible increase in the rates of denitrification as the crop was aging, which can be ascribed to the gradual increase in the air temperature, given that the crop was established on 16 January and the commencement of the reproductive stage coincided with the advent of the spring season. Nitrogen losses through denitrification in hydroponic NSs may be considerable, reaching levels up to 20% of the total supply, as has been shown by Daum and Schenk (1998). According to the method applied in the present study to estimate UC, denitrification losses are taken as apparent N uptake and, therefore, they tend to increase the estimated UC.

Since the UC represent uptake ratios between a nutrient and water, significant differences in the UC of any nutrient between cultivars originate from differences either in whole plant transpiration owing to leaf area differences, or in the nutrient demand, or in a combination of them. Furthermore, any differences in the nutrient demand between cultivars are mainly due to commensurate differences either in tissue nutrient concentrations or in the fruit biomass. The significantly higher Ca and Mg UC by Sondela and K UC by Bellisa in comparison with those recorded in all other genotypes, which were consistent during the whole cropping period, are partly ascribed to lower whole plant transpiration (**Figure 1**). ‘Sondela’ and ‘Bellisa’ are cultivars grown for the production of red pepper fruits, which need longer time to ripen than those harvested green, as is the case with ‘Sammy.’ It is well known that the delay in fruit harvesting, which increases the fruit load, retards the vegetative growth (Engels et al., 2012), thereby reducing the leaf area per plan and concomitantly the whole plant transpiration. Nevertheless, the selective increase of only the UC of Ca and Mg in Sondela and K in Bellisa suggests that not only the whole plant transpiration but also the nutrient requirements are different between different cultivars. Indeed, while the whole plant transpiration was lower in both ‘Sondela’ and ‘Bellisa’ in comparison to the other genotypes, the leaf Ca and Mg concentrations were higher only in ‘Sondela’ during the reproductive stage of development. Hence, the factor that ultimately imposed higher Ca and Mg UC in ‘Sondela’ than in the other genotypes was the higher concentration of Ca in the leaves, and Mg in both leaves and fruit of ‘Sondela’ (**Figures 2B, 3B**). The reduced Ca and Mg concentrations in the NS that was recirculating in circuits accommodating ‘Sondela’ are in agreement with the higher Ca and Mg UC found for this cultivar. The same applies also for K in ‘Bellisa.’ The significantly higher K UC in ‘Belisa’ were also due to a combination of higher whole plant transpiration and higher leaf K concentrations as indicated by **Figures 1 and 4A**.

The UC of total-N and P do not seem to be influenced by the genotype of the cultivar. Finally, the UC of the micronutrients Fe, Mn, Zn, and B did not show a consistent influence of the genotype, although ‘Sondela’ exhibited significantly higher Zn and B UC than the other cultivars at certain developmental stages. Sondela exhibited higher boron UC only during the reproductive developmental stage, which points to an effect of the fruit load on boron uptake rather than a transpiration-related effect on B UC.

An appreciable body of related investigations has indicated that grafting may decrease the uptake of some nutrients while increasing the uptake efficiency for some other nutrients depending mainly on the rootstock genotype (Ruiz et al., 1997; Rouphael et al., 2008; Savvas et al., 2009; Colla et al., 2010). Therefore, grafting has been suggested as a means to limit nutrient and heavy metal toxicity, or to increase fertilizer use efficiency and prevent nutrient deficiencies in marginally fertile soils (Savvas et al., 2013; Rouphael et al., 2016). Many rootstocks used to graft vegetables are wild genotypes of the same species as the scion, relatives, or hybrids of them, which are characterized by more vigorous root systems than those of highly productive cultivars (Huang et al., 2010; Pico et al., 2017). However, the pepper rootstock RS10, which was used to graft ‘Sammy’ in the current research, belongs to the same species with the scion (*Capsicum annuum*) and not to any wild relative species of paper characterized by a vigorous root system. This is presumably the reason for the lack of any differences in leaf and fruit nutrient levels as well as in UC between self-grafted ‘Sammy’ plants and ‘Sammy’ plants grafted onto the rootstock RS10.

Comparing the UC found in the present study with those found by Voogt and Sonneveld (1997) in North European hydroponic greenhouses reveals that they are influenced by the contrasting climatic conditions. Indeed, the UC found by Voogt and Sonneveld (1997) in a pepper crop grown in a closed rockwool system in the Netherlands, averaged for the whole cropping period, were 2.2 mmol L^-1^ for Ca, 0.78 mmol L^-1^ for Mg, 4.6 mmol L^-1^ for K, 10.30 mmol L^-1^ for N, and 0.81 mmol L^-1^ for P. The corresponding UC found in the present study ranged from 2.4 to 3.7 mmol L^-1^ for Ca, 1.0 to 1.5 mmol L^-1^ for Mg, 6.1 to 9 mmol L^-1^ for K, 11.7 to 13.7 mmol L^-1^ for N, and 0.7 to 1.1 mmol L^-1^ for P. A comparison between the values found in the two different studies reveals that the UC of macronutrients found in the present study are clearly higher than those reported by Voogt and Sonneveld (1997). In a similar study (Savvas et al., 2017), tomato plants grown under Mediterranean climatic conditions had an increased need for N, P, K, Ca, Zn, and Cu and a decreased need for Fe, Mn, and B in comparison with the UC reported under North European climatic conditions (Sonneveld and Voogt, 2009). The differences in UC between greenhouses located in these two contrasting environments may originate from differences either in the mean light interception, as reported by Adams (1993), or in the method applied to determine the UC, as reported by Tzerakis et al. (2013), Neocleous and Savvas (2015), and Savvas et al. (2017). The method applied in the present study to determine the UC takes into consideration possible precipitation losses. Therefore, the obtained values should be correctly termed “apparent UC” (Adams, 2002). However, for a sufficient supply of nutrients to plants grown in CHS, it is essential to provide the amount of nutrients corresponding not only to net uptake but also to losses through precipitation or immobilization that may occur during the flow of the NS along the system. Therefore, the apparent nutrient UC determined in the present study constitute a sound basis to establish suitable NS compositions for pepper crops cultivated in CHS under Mediterranean climatic conditions.

## Conclusion

The results of the present study indicated that different pepper cultivars may take up nutrients and water at different ratios under the same nutritional, irrigation, and climatic conditions, as indicated by the observed differences in the UC. More specifically, ‘Sondela’ exhibited the highest Mg and Ca UC throughout the cropping period and B UC during the reproductive stage in comparison with all other tested cultivars. Furthermore, ‘Bellisa’ exhibited significantly higher K UC throughout the cropping period in comparison with all other tested cultivars.

The tissue nutrient concentrations and the UC were similar in ‘Sammy’ self-grafted and ‘Sammy’ grafted onto the commercial rootstock ‘RS10’ (*Capsicum annuum*), which indicates that this *Capsicum annum* rootstock does not modify the uptake of nutrients and water by the scion. The developmental stage of the pepper plants had a strong impact on the UC of most nutrients and this is ascribed to changes in the mean physiological age of leaves and differences in fruits load. Based on the UC estimated in the present study, the NSs supplied to closed hydroponic crops of pepper should contain Ca, Mg, and B at higher concentrations than standard recommendations when the cultivated variety is ‘Sondela’ and K at higher concentrations when the cultivated variety is ‘Bellisa.’

## Author Contributions

GN, CK, NK, and DS conceived and designed the experiments. AR and GN performed the experiments. AR, GN, NK and DS analyzed the data and wrote the paper. CK, NK, and DS reviewed the paper. All authors have read and approved the manuscript.

## Conflict of Interest Statement

The authors declare that the research was conducted in the absence of any commercial or financial relationships that could be construed as a potential conflict of interest.
